# 3D Automated Segmentation of Lower Leg Muscles Using Machine Learning on a Heterogeneous Dataset

**DOI:** 10.3390/diagnostics11101747

**Published:** 2021-09-23

**Authors:** Marlena Rohm, Marius Markmann, Johannes Forsting, Robert Rehmann, Martijn Froeling, Lara Schlaffke

**Affiliations:** 1 Department of Neurology, BG-University Hospital Bergmannsheil gGmbH, Ruhr-University Bochum, 44789 Bochum, Germany; marius.markmann@rub.de (M.M.); johannes.forsting@rub.de (J.F.); robert.rehmann@rub.de (R.R.); lara.schlaffke@rub.de (L.S.); 2 Heimer Institute for Muscle Research, BG-University Hospital Bergmannsheil gGmbH, 44789 Bochum, Germany; 3 Department of Neurology, Klinikum Dortmund, University Witten-Herdecke, 44137 Dortmund, Germany; 4 Department of Radiology, University Medical Centre Utrecht, 3584 Utrecht, The Netherlands; m.froeling@umcutrecht.nl

**Keywords:** qMRI, muscle segmentation, machine learning

## Abstract

Quantitative MRI combines non-invasive imaging techniques to reveal alterations in muscle pathophysiology. Creating muscle-specific labels manually is time consuming and requires an experienced examiner. Semi-automatic and fully automatic methods reduce segmentation time significantly. Current machine learning solutions are commonly trained on data from healthy subjects using homogeneous databases with the same image contrast. While yielding high Dice scores (DS), those solutions are not applicable to different image contrasts and acquisitions. Therefore, the aim of our study was to evaluate the feasibility of automatic segmentation of a heterogeneous database. To create a heterogeneous dataset, we pooled lower leg muscle images from different studies with different contrasts and fields-of-view, containing healthy controls and diagnosed patients with various neuromuscular diseases. A second homogenous database with uniform contrasts was created as a subset of the first database. We trained three 3D-convolutional neuronal networks (CNN) on those databases to test performance as compared to manual segmentation. All networks, training on heterogeneous data, were able to predict seven muscles with a minimum average DS of 0.75. U-Net performed best when trained on the heterogeneous dataset (DS: 0.80 ± 0.10, AHD: 0.39 ± 0.35). ResNet and DenseNet yielded higher DS, when trained on a heterogeneous dataset (both DS: 0.86), as compared to a homogeneous dataset (ResNet DS: 0.83, DenseNet DS: 0.76). In conclusion, a CNN trained on a heterogeneous dataset achieves more accurate labels for predicting a heterogeneous database of lower leg muscles than a CNN trained on a homogenous dataset. We propose that a large heterogeneous database is needed, to make automated segmentation feasible for different kinds of image acquisitions.

## 1. Introduction

Quantitative magnetic resonance imaging (qMRI) provides promising surrogate bio-markers in the evaluation of disease progression and monitoring of therapeutic options in neuromuscular diseases (NMD) [1,2]. This non-invasive technique can reveal subclinical changes in muscle pathophysiology which can precede changes in muscle function assessed by clinical examination [3]. In NMD different patterns of muscle involvement have been described and are essential to distinguish between different subtypes of diseases [4,5]. Therefore, muscle segmentation plays a key role in the analysis of qMRI data.

So far, the segmentation of individual muscles has to be performed manually as there are no generalizable fully-automatic algorithms available yet [6]. Manual segmentation is very time consuming and especially highly degenerated and fat-infiltrated muscles lead to difficulties in separation of muscle groups. Manual segmentation is a bottleneck, and therefore a major limitation in the application of qMRI in clinical studies. This has driven researchers towards developing automated solutions using algorithmic machine learning solutions [7]. Defining each muscle separately and segmenting an image into n labels can be framed as a categorization problem where the goal is to find the right category for each voxel in the image. Early research in this field has often used classification algorithms such as random walk [8] or random forest [9]. Both attempts resulted in high congruency between manual and automated classification, but their approaches were limited by their ability to transfer to other image contrasts or when segmenting patient data with fatty degeneration or muscle atrophy. As both, random walk and random forest algorithms do not construct explicit edge detectors, their ability to generalize to fatty muscle data is impeded. Other approaches to segment data for quantitative analysis are deep learning-based solutions such as convolutional neural networks (CNN) architectures. These algorithms often outperform classical machine learning algorithms, without manual feature selection [10].

We compare three fundamental 3D-CNN architectures in this paper. U-Net architectures are often used as a comparative baseline for other network architectures [11]. They have been widely used and adapted to clinical applications from detecting skin lesions [12], parotid glands [13], pulmonary nodules [14], segmented infant-brain MR-images [15], cardiac segmentation [16], as well as cell structures in light microscopy images [17]. ResNet architectures use residual connection and allow blocks to learn residual functions. Theoretical discussion in machine-learning has argued that residual connections allow networks to learn faster, and generalize better [18,19]. ResNet blocks also allow the network to train deeper network architectures without facing the problem of vanishing gradient [20]. ResNet architectures have recently been adapted to medical image segmentation [21] and have been used to segment tongue compartments [22]. Although they have significant theoretical advantages over U-Nets, they have been applied less in medical image segmentations. Finally, DenseNets are the most recent architectures among the three major CNN designs discussed here. Similar to ResNets, they use residual connections that allow DenseNets to learn and generalize the same way [23], but instead of increasing network depth, they retain almost all information between layers. This allows networks to reuse features from earlier layers, but also drastically increases memory requirements as the amount of feature maps processed by later layers grows linearly with network depth. DenseNets have been shown to be successful in classical image segmentation tasks [24], as well as brain tumor segmentation [25]. 

Previous work in automated muscle segmentation is limited by small sample sizes or the homogeneity of the datasets. To allow this method to be applicable for a broad range of appliances, a CNN trained on different image contrasts and data from different disease types is needed. So far, there is no method that can be used independent of the imaging protocol and has been validated for various muscle disease types. Our aim was to show that convoluted networks are able to generalize over large variations in both data acquisition and health status of the patient. Therefore, the feasibility of CNNs-U-Net, ResNet and DenseNet was evaluated to segment muscles when trained on a heterogeneous as compared to a heterogeneous dataset with varying acquisition protocols and imaging contrasts. 

## 2. Materials and Methods

### 2.1. Datasets

**Figure 1:**
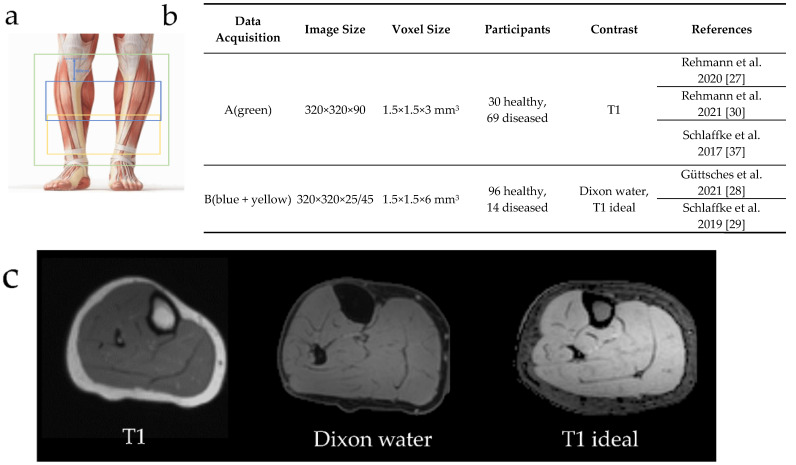
Overview of the diversity of MR-images included in the database. **a:** The data acquisitions differ in their field-of-view. The green rectangle represents a field-of-view for data acquisition (A) with 90 slices in the z-axis, while the blue and yellow rectangles (B) show the 25 and 45 slices from data acquisition, respectively, measured 60 cm ventral from the tibial plateau. **b:** Comparison between data acquisition (A) and (B) regarding image size, voxel size, participants and contrasts. **c:** Three different types of contrasts are included in the database.

MR-images of lower leg muscles from 126 healthy controls and 83 diagnosed patients were included. This database was pooled from data of different studies [26,27,28,29,30], conducted according to the guidelines of the Declaration of Helsinki and approved by the ethic committee of the medical faculty of the Ruhr-University Bochum (15-5281, 31 March 2015). Informed written consent was obtained from all subjects. The database can be divided into two different data acquisitions protocols mainly distinguishable due to their different fields-of-view (FOV) and contrasts (Figure 1). In data acquisition (A) the whole lower leg was covered using 90 slices. In data acquisition (B) only 25 or 45 slices were measured 60 cm ventral from the tibial plateau. Both datasets included patient data covering various muscular diseases: Morbus Pompe (*n* = 22), McArdle (*n* = 8), inclusion-body myositis (IBM, *n* = 6), myotonic dystrophy type 1 (MDI, *n* = 10), myotonic dystrophy type 2 (MDII, *n* = 13), leg-girdle muscular dystrophy (LGMD, *n* = 6) and others (*n* = 18).

Acquisition (B) contained MR-images of a multicenter study and is called the homogeneous dataset in the following [29]. This database included 93 datasets from healthy participants.

### 2.2. Manual Segmentation

Manual segmentation was performed using 3D Slicer (4.4.0, https://slicer.org accessed on 1 March 2021) by an expert with 5 years of experience (author: MR). Based on a T1w contrast (acquisition (A)) or a Dixon water image (acquisition (B)) and by avoiding subcutaneous fat and fascia, these muscles were manually segmented on all slices. Adjacent muscles with high fatty infiltration were separated by considering anatomical features. The segmentation produced labels covering the following seven lower leg muscles: (1) gastrocnemius lateralis, (2) gastrocnemius medialis, (3) soleus, (4) tibialis anterior, (5) peroneus, (6) extensor digitorum longus + extensor hallucis longus, (7) tibialis posterior.

### 2.3. Data Selection and Composition

**Figure 2:**
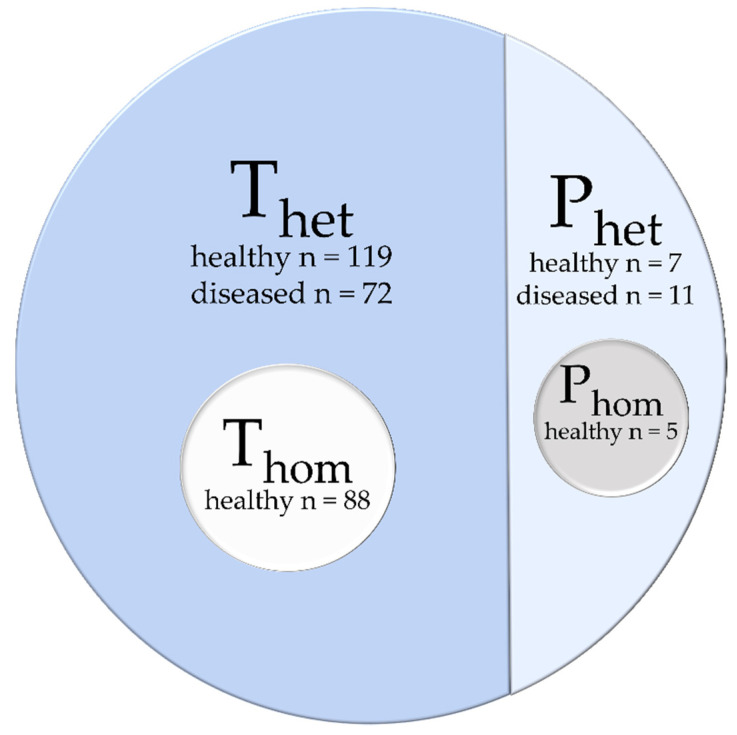
The database is distributed into two main parts: a heterogeneous training dataset T_het_ and a prediction dataset P_het_. A homogeneous training dataset T_hom_ and prediction dataset P_hom_ are taken as a subset from the heterogeneous datasets.

The database was distributed into four different datasets (Figure 2): (i) a heterogeneous dataset used for training (T_het_), contained images and labels from 119 healthy participants and 72 diseased patients of both data acquisitions (A) and (B), (ii) a homogenous training dataset (T_hom_) included images and labels from 88 healthy participants from data acquisition (B), (iii) a heterogeneous dataset for prediction with images of seven healthy subjects and eleven diseased patients, (iv) a homogeneous dataset (P_hom_) with five of the in P_het_ included healthy subjects. 

### 2.4. Preprocessing

Three-dimensional (3D) MR-images of the lower leg were collected from different studies and differed in their contrast, slice thickness and positioning of the FOV. CNNs only take in one input size. To comply to this and reduce memory usage a universal preprocessing was used for those 3D images with their respective labels. First, each image was split on the *z*-axis into parts of 20 to 21 slices and then range-normalized from 0 to 100. Since processing the segmentation is equivalent for left and right leg, the images and their labels were split and then the left leg was mirrored. The background of each image and label was cut automatically to reduce memory usage. Then, all images and labels were interpolated to result in 3D arrays in the same dimension (104 × 104 × 20). As a last step, the manual segmented labels were one-hot encoded (n = 8, each muscle plus background).

### 2.5. Postprocessing

All predicted labels were postprocessed to result in a label fitted on the original images. Networks computed probability distributions as vectors of length eight for each voxel. Labels were extracted by assigning the class with the highest probability for each voxel. Then the dimensions were restored with interpolation and background padding. The left leg was mirrored and merged onto the right leg. For original images with more than 25 slices, an overlap of three slices was created during preprocessing and resolved during postprocessing.

### 2.6. Convolutional Neuronal Networks

We compared three different neural network architectures with respect to their ability to adapt to homogeneous and heterogeneous datasets. All networks were implemented using 3D Convolutional Networks. 

The 3D-U-Net by Çiçek et al., 2016 [31] is based on the 2D-U-Net-Model by Ronneberger et al., 2015 [11]. They proposed a structure with contracting and expanding pathways and identity skip-connections between both pathways. We used maxpooling for downsampling and transposed convolutions for upsampling. Our implementation of U-Net had 16,259,464 free parameters and required a GPU with 8.00 Gb of VRam for training. 

The second network we tested was a ResNet with a contracting and expanding pathway like U-Net. It was proposed by Drozdzal et al., 2016 [21]. Their architecture integrates ResNet blocks into the contracting and expanding pathways. Downsampling was done via convolutions using kernels with size = 1 and strides = 2, upsampling was done by repeating voxels per dimension. Our implementation of ResNet had 32,924,968 free parameters and required a GPU with 7.47 Gb of VRam for training. 

Finally, we used a DenseNet implementation proposed by Jegou et al. [24], which followed the basic contraction and expansion pathway, but implements DenseBlocks similar to those proposed by Huang et al. [23] as layer modules. Our implementation of DenseNet had 1,727,256 free parameters and required a GPU with 21.91 Gb of VRam for training. 

All Networks were implemented in Keras (Version 2.3.1) on a Tensorflow (2.1.0) backend. They were trained on an RTX6000 graphics card using Adam [30] with a learning rate of 1 × 10^−5^ and a decay of 1.99 × 10^−6^. Learning was optimized to reduce categorical cross entropy. The networks were trained for 200 epochs with a batch size of six, after which none of the networks showed improvement in performance. The networks were optimized for categorical cross entropy, with a softmax activation function as an output layer. Both training datasets were shuffled once before training and then split into a train and validation set with proportions of 80 and 20 percent. Both sets were kept identical between networks to ensure comparability but were shuffled after each epoch. 

### 2.7. Evaluation

For comparing predicted 3D-labels to manual segmentation, we used the open-source software VISCERAL EvaluateSegmentation [32]. Dice score (DS) as a marker for 3D overlap and average Hausdorff distance (AHD) as a marker of average distance between predicted and manually labelled muscle borders defined in mm were used to characterize and compare the performance of each model. DS being best for a value of 1 and AHD being best for a value of 0. The statistical evaluation was done in R (4.0.3). For statistical comparison all scores were averaged over muscles per image.

We compared model performance for predicting on images similar to the dataset they were trained on (T_het_/P_het_, T_hom_/P_hom_) as well as for predicting the dataset they were not trained on (T_hom_/P_het_, T_het_/P_hom_). To compare the effect of a heterogeneous or homogeneous dataset for training, the DS and AHD for predicting a homogeneous dataset were statistically evaluated (T_het_/P_hom_, T_hom_/P_hom_). In order to analyze network performance predicting patients, we pooled all patient-images, already contained in P_het_, and predicted them with all three networks trained on the heterogeneous and homogeneous dataset.

## 3. Results

U-Net, ResNet and DenseNet were each trained on a homogeneous (T_hom_) and a heterogeneous dataset (T_het_). After 200 epochs an independent dataset, containing 3D MR-images from the same data acquisition as the homogeneous dataset (P_hom_) as well as others (P_het_), was used for prediction of muscle segmentations and evaluation. 

**Figure 3:**
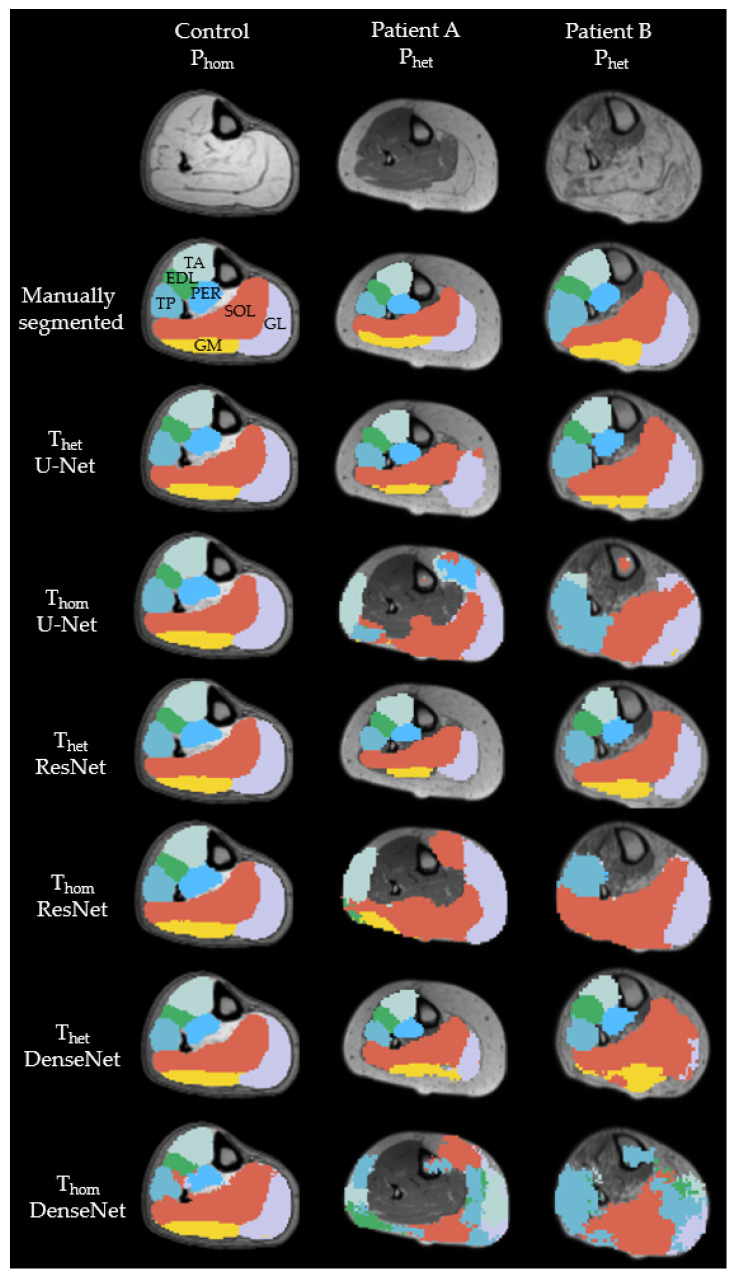
Examples of cross-sectional segmentation overlaid with MR-images of one representative healthy control and two different patients. Each column represents a different network, trained on either a heterogenous or homogenous dataset. EDL = extensor digitorum longus (green); GL = gastrocnemius lateralis (purple); GM = gastrocnemius medialis (yellow); PER = peroneal group (light blue); SOL = soleus (orange); TA = tibialis anterior (mint); TP = tibialis posterior (blue).

Figure 3 shows cross-sections of representative MR-images of a healthy subject, as representation for P_hom_, and two patients, as representation for P_het_. The labels were predicted with U-Net, ResNet and DenseNet trained on T_het_ and T_hom_ respectively. All seven labels were present for all models, when trained on T_het_ predicting on P_het_ as well as trained on T_hom_ and predicting on P_hom_. For the control image, all networks were able to predict the shape of all muscles correctly, but DenseNet trained on T_hom_ predicted parts of the soleus into the border between tibialis posterior and peroneus. Results from a representative patient A show that both ResNet and DenseNet trained on T_het_ were able to detect muscle borders and locations correctly. When comparing predictions for the gastrocnemius lateralis, we see that all networks trained on T_hom_ and the T_het_ trained U-Net were unable to segment the border between subcutaneous fat and muscle. Both ResNet and DenseNet trained on T_het_ predicted shapes similar to the manual segmented image. Finally, labels for Patient B show that networks trained on T_het_ were able to reproduce the general shape of the muscles with DenseNet failing to build consistent edges for both gastrocnemius medialis and gastrocnemius lateralis. For DS and AHD of each muscle, model and scenario see Appendix A.

**Figure 4:**
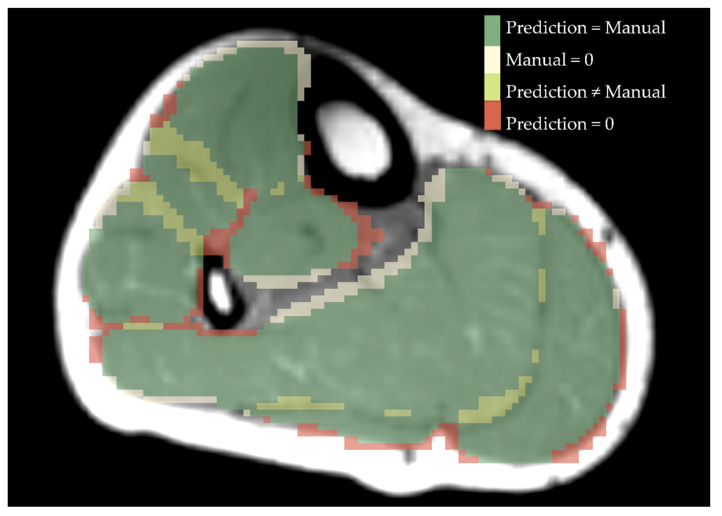
Overlay of manual segmentation and a label predicted by ResNet for a healthy control. Green shows the area that is labelled equally, beige is predicted as a specific muscle but was not drawn manually, yellow are different numbers of labels drawn and predicted and red displays what was drawn manually but not predicted by ResNet.

As an example, Figure 4 shows an overlay of a manually segmentation and a ResNet-predicted-label.

An overview of all DS as a marker for 3D similarity and AHD as a marker of average distance between predicted and manually labeled muscle borders is shown in Table 1.

For T_het_ and P_het_ all three models performed similarly to each other, with DenseNet providing the highest DS (DS: 0.81 ± 0.09) followed by U-Net (DS: 0.80 ± 0.10) and ResNet (DS: 0.79 ± 0.10). DenseNet had a significantly higher DS than ResNet (t(35) = 2.44, *p* = 0.02). U-Net (AHD: 0.39 ± 0.37) had a significantly lower AHD than ResNet (AHD: 0.43 ± 0.35) (t(35) = 2.525, *p* = 0.016). All other comparisons yielded no significant results.

When evaluating T_hom_ and P_hom_, all scores were significantly different from each other with U-Net providing the highest DS and lowest AHD (DS: 0.86 ± 0.07, AHD: 0.26 ± 0.25). DenseNet (DS: 0.76 ± 0.09, AHD: 0.66 ± 0.39) performed significantly worse than U-Net (DS: t(9) = 8.87, *p* < 0.001, AHD: t(9) = 6,78, *p* < 0.001) and ResNet (DS: 0.83 ± 0.07, AHD: 0.35 ± 0.29) (DS: t(9) = 3.104, *p* < 0.001, AHD: t(9) = 2.75, *p* < 0.001).

**Figure 5:**
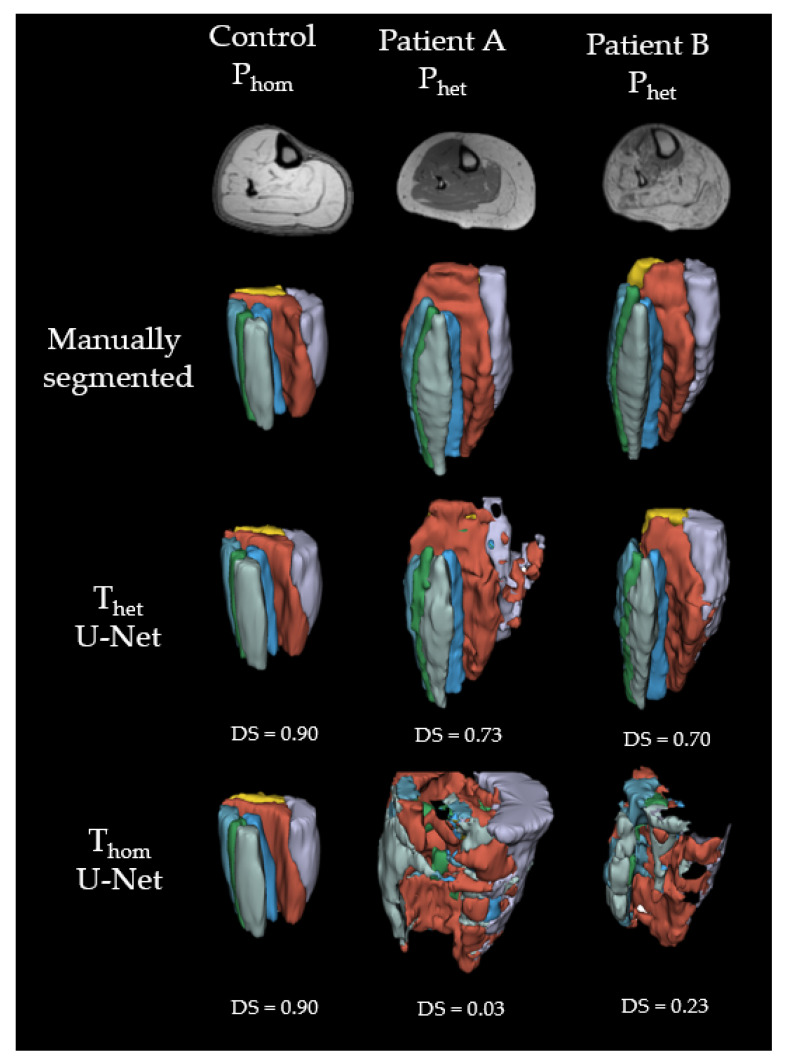
Examples of 3D labels predicted with U-Net. Green = extensor digitorum longus (EDL); purple = gastrocnemius lateralis (GL); yellow = gastrocnemius medialis (GM); light blue = peroneal group (PER); orange = soleus (SOL); mint = tibialis anterior (TA); blue = tibialis posterior (TP).

As seen in Figure 5, all three CNNs trained on T_hom_ and predicted on P_het_ were not able to localize the seven muscles in a non-familiar contrasted image. When predicting P_het_, U-Net and ResNet trained with T_hom_ yielded the same mean DS (U-Net: 0.38 ± 0.35, ResNet: 0.38 ± 0.36). DenseNet (DS: 0.29 ± 0.34, AHD: 12.2 ± 9.60) was significantly worse than ResNet (DS: 0.38 ± 0.35, AHD: 7.24 ± 5.67) in DS (t(9) = 6, *p* < 0.001)) and AHD (t(9) = 5.88, *p* < 0.001).

There were no significant differences between models when trained on T_het_ and predicted on P_hom_.

Comparing network performance predicting P_hom_ being trained on either T_het_ or T_hom_ we found a significant difference for ResNet and DenseNet. DenseNet trained on T_het_ (DS: 0.86 ± 0.05, AHD: 0.25 ± 0.21) shows significantly higher DS and lower ASD compared to T_hom_ (DS: 0.76 ± 0.09, AHD: 0.66 ± 0.39) (DS: t(9) = 7.28, *p* < 0.001, AHD: t(9) = 6.59, *p* < 0.001). ResNet trained on T_het_ (DS: 0.86 ± 0.06, AHD: 0.26 ± 0.22) shows significantly higher DS and lower ASD in comparison to T_hom_ (DS: 0.83 ± 0.07, AHD: 0.35 ± 0.29) (DS: t(9) = 3.68, *p* = 0.005, AHD: t(9) = 2.54, *p* = 0.03). There were no significant differences for U-Net.

**Figure 6:**
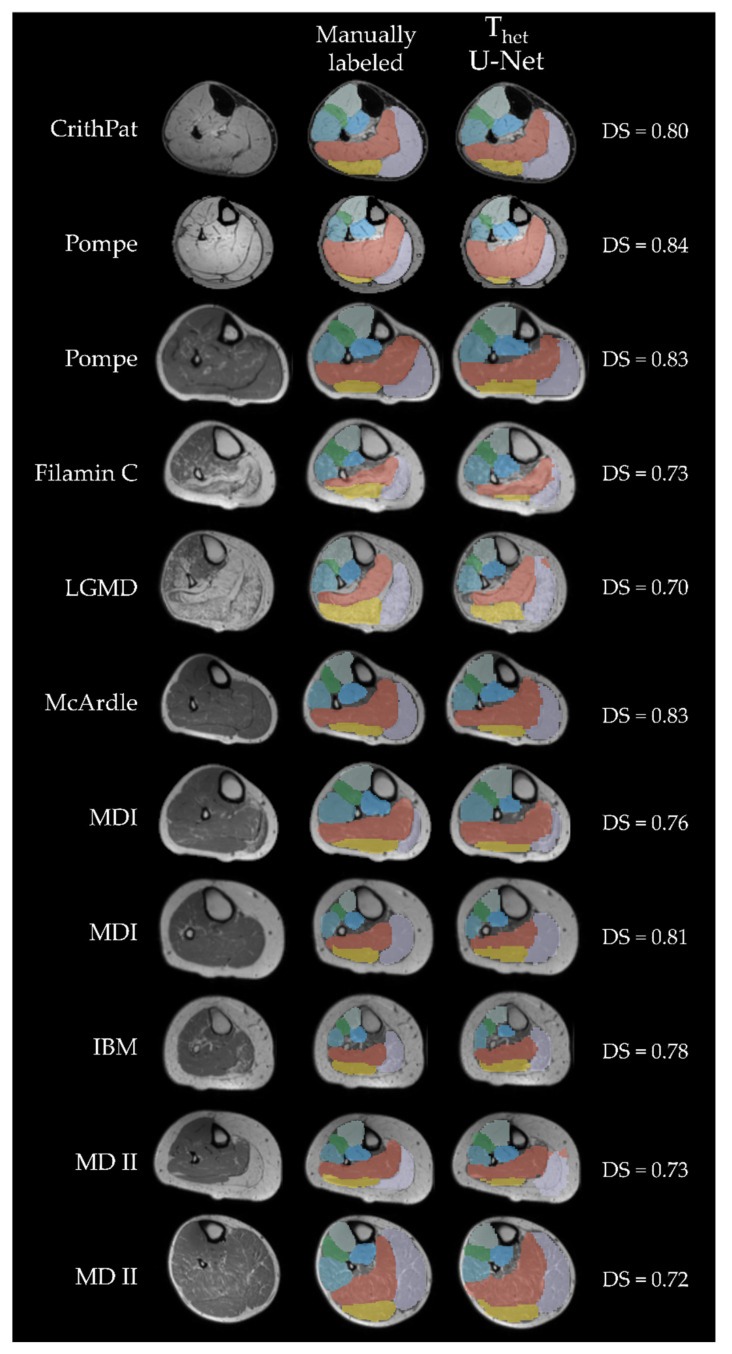
All cross-sectional patient MR-images included in P_het_. Each column displays a patient, while each row shows the MR image, manual segmentation and U-Net predicted label, respectively. The first two rows show Dixon-weighted-images from data acquisition (B), all others are T1 weighted images from data acquisition (A). Labels are displayed with transparency to see underlying boundaries. Green = extensor digitorum longus (EDL); purple = gastrocnemius lateralis (GL); yellow = gastrocnemius medialis (GM); light blue = peroneal group (PER); orange = soleus (SOL); mint = tibialis anterior (TA); blue = tibialis posterior (TP); CrithPat = critical illness polyneuropathy; Pompe = Morbus Pompe; Filamin C = filamin C myopathy; LGMD = leg-girdle muscular dystrophy; MDI = myotonic dystrophy type 1; IBM = inclusion-body myositis; MDII = myotonic dystrophy type 2.

Finally, we compared DS and AHD for all networks trained on T_het_ and only predicting patient data. As an example, Figure 6 displays cross-sections of all patient images contained in P_het_ predicted with U-Net. DenseNet (DS: 0.79 ± 0.06) had a significantly higher DS than ResNet (DS: 0.77 ± 0.05) (t(25) = 2.17, *p* = 0.039). U-Net (DS: 0.78 ± 0.05) had a significantly higher DS than ResNet (DS: 0.77 ± 0.05) (t(25) = 2.84, *p* = 0.009). Finally, we found a significant difference in AHD between ResNet (AHD: 0.26 ± 0.20) and U-Net (AHD: 0.26 ± 0.21) (t(25) = 2.61, *p* = 0.015).

## 4. Discussion

We were able to show that different CNNs are able to learn to segment lower leg muscles in MRI image data. U-Net performs best when the database is homogenous and DenseNet and U-Net outperforming ResNet in cases where networks were trained on a heterogenous database. Both DenseNet and U-Net performed well when trained on T_het_ and predicted on P_het_. Finally, as shown in Figure 3, all three models were able to learn muscle borders and locations, independent of the respective image contrast.

Given the differences in performance, in respect to the different training and prediction datasets there seems to be no global gold standard, but recommendations for different applications. With identical contrasts and a homogeneous database, even a simple U-Net structure was able to outperform both ResNet and DenseNet in both scores. Given a more complex task in terms of image variance both DenseNet and U-Net were able to predict data from different acquisition protocols and patient groups. This implies that in studies with healthy participants any of the three network architectures can be used. Studies on patient data, or data with more intrinsic variance will profit greatly from a pretrained network trained on a large heterogeneous database.

Our aim was to make steps towards a tool able to segment MR-images in the lower leg capable of handling images acquired on different scanners measuring different contrasts and patient groups. For small or homogenic training datasets image augmentation could be used to rotate or shift images, allowing CNNs to adapt to variances. For this study we limited data augmentation to range normalization to show the influence of a heterogenic dataset, with MR-images pooled from different studies and even different scanners [29]. This heterogeneous information can help the network to build generalizable representations which was shown in our study by the slight but significant performance increase in DS for ResNet and DenseNet predicting P_hom_ when trained on T_het_ compared to T_hom_. This implicates that a large heterogeneous database for training would improve segmentation quality, even for tasks where the expected variance is comparatively low. When trained on T_het_ AHD significantly increased when networks were also trained using patient data, compared to using data from healthy participants only. This indicates that even when networks were able to locate muscles correctly, they had issues with detecting muscle boundaries in patient images. A recent work by Guo et al., 2021 has addressed this issue by integrating self-learning edge gates into their network, significantly increasing network performance [33]. Integrating self-learning edge gates into existing network architectures would likely increase performance for data where muscles contain a higher fat fraction or when the training database contains data from different acquisition protocols.

Another important point is to include MR-images with different FOVs to be able to predict the labels no matter which region of the leg was scanned. Full-leg MRI scans lead to a huge amount of information, but also to an enormous time investment in acquisition and segmentation of all muscles. In data acquisition (A) the FOV was set irregular, sometimes reaching into the knee or in the ancle depending on the subject’s height. Training on all data, all CNNs shown here were able to predict the labels for MR-images of the lower leg reaching into the knee or ancle. This shows that a heterogeneous dataset, regarding different FOVs, improves prediction quality and diminishes the factor of a trained CNN being only usable for similar data with uniform FOV positioning.

Finally, we show that the CNNs used here are able to predict labels for various diseases, such as Morbus Pompe, IBM, LGMD and others. It is important to cover as many disease groups as possible, because different diseases show different patterns of affected muscles and therefore different muscle borders become harder to detect [5,34]. In addition to that, atrophy leads to changes in morphology of single muscles which impacts the localization of other muscles [35]. P_het_ contained MR-images of eleven patients with several different NMDs. The differences in morphology and fat infiltration can be observed in Figure 6. Training on this range of different types of NMDs, improves the resulting labels of a CNN for fat infiltrated images of patient data. Using a diverse cohort for training makes the CNN prone to also predict images of patients with diseases that were not used for training in this study, such as Duchenne muscular dystrophy (DMD).

Till now, all proposed muscle segmentation algorithms lack in accuracy, when segmenting muscles from patients with muscle fat depositions and fibrosis. As we can see from Figure 3, the performance of muscle segmentation for patients with high amount of fatty infiltration was lower. However, all networks trained on T_het_ predicted muscle shapes correctly, with slight errors on the edge of the muscle. When borders between muscles and subcutaneous fat have disintegrated, as in the patient B from Figure 3, all networks fail to correctly assign muscle borders. This is particularly visible for the border between the gastrocnemius medialis and the surrounding gastrocnemius lateralis, soleus and subcutaneous fat. All networks trained on T_het_ have problems to recreate the border shape, by either falsely defining a precise border between gastrocnemius medialis and soleus, as DenseNet does, or wrongly labeling parts of the muscle as background, as ResNet and U-Net do. Interestingly, patient A shows less fat infiltration than patient B, but since the fat is only affecting one muscle and making it look such as subcutaneous fat, U-Net fails to draw a precise boundary. This can be seen best in the 3D label in Figure 5. Adapting more complex edge detection algorithms could mitigate these errors in future research. One limit of describing predictions of CNNs is to compare them to manual segmentation, which is seen as the gold standard. Since manual segmentation is time consuming, time pressure as well as software tools might reduce the accuracy of drawn labels. The better the quality of the manual annotation used for training the better the results. However, achieving constant quality of segmentations, especially concerning the small details, can be very difficult and even more time consuming as generating segmentations for data analysis. Furthermore, for voxels that are on the border to subcutaneous fat it is difficult to decide if they should be labeled to a specific muscle or not. However, most post-processing steps involve smoothing and erosion of the labels to diminish partial volume effects [29]. When overlaying manually segmented and with CNNs predicted labels, it is obvious that some variance is due to the areas close to subcutaneous fat, as seen in Figure 4. This variance is tolerable but is leading to a lower DS. While the predicted labels show some spuriously classified voxels, the here presented labels can be manually refined to decrease segmentation time compared to full manual segmentation. In addition to that, the accuracy of the volume might not be the most important factor when analyzing clinical parameters such as fat fraction or diffusion parameters. A promising approach already showed diffusion parameters to be consistent comparing manual segmentation and semi-automated segmentation on the upper leg [36]. An interesting question for future studies would be to see the needed accuracy of a 3D labeling technique when analyzing clinical parameters [37].

## 5. Conclusions

Our results provide evidence that using a heterogeneous training dataset an automated unified solution can be used for muscle segmentation, with varying image contrasts, and for FOVs or health status of the participants. The CNNs ability to generalize to new data is dependent on the heterogeneity of the database. Our data suggest that a global database from various scanners and sides is desirable. To conclude, we were able to show that CNNs will be able to remove the time consuming bottleneck from qMRI analysis, paving the way to apply qMRI data acquisition in a clinical routine as a non-invasive surrogate biomarker.

## Figures and Tables

**Table 1:** Overview of Dice scores and average Hausdorff distances for all three CNN predicted labels compared to manually segmented labels with different training and prediction datasets. Dice score is best for a value of 1 and AHD for a value of 0.

**Predicting** **Training**		**Dice Score**						**Average Hausdorff Distance**					
		**Homogeneous** **P_hom_**			**Heterogeneous** **P_het_**			**Homogeneous** **P_hom_**			**Heterogeneous** **P_het_**		
		**Mean ± SD**			**Mean ± SD**			**Mean ± SD**			**Mean ± SD**		
Homogeneous T_hom_	U-Net	0.86	±	0.07	0.38	±	0.36	0.26	±	0.25	7.98	±	6.57
	ResNet	0.83	±	0.07	0.38	±	0.35	0.35	±	0.29	7.24	±	5.67
	DenseNet	0.76	±	0.09	0.29	±	0.34	0.66	±	0.39	12.2	±	9.60
Heterogeneous T_het_	U-Net	0.85	±	0.08	0.80	±	0.10	0.26	±	0.23	0.39	±	0.37
	ResNet	0.86	±	0.06	0.79	±	0.10	0.26	±	0.22	0.43	±	0.35
	DenseNet	0.86	±	0.05	0.81	±	0.09	0.25	±	0.21	0.41	±	0.40

## Data Availability

Data used in our study are available on request but cannot be made public due to data privacy concerns.

## Appendix A

**Table A1:** Overview of mean Dice score and average Hausdorff distance calculated by muscle.

Predicting Training			Dice Coefficient		Average Hausdorff Distance	
			Homogeneous P_hom_ Mean ± SD	Heterogeneous P_het_ Mean ± SD	Homogeneous P_hom_ Mean ± SD	Heterogeneous P_het_ Mean ± SD
Homogeneous T_hom_	U-Net	GM	0.92 ± 0.04	0.46 ± 0.36	0.14 ± 0.16	4.21 ± 4.21
		GL	0.81 ± 0.15	0.34 ± 0.36	0.39 ± 0.49	5.26 ± 4.88
		SOL	0.85 ± 0.12	0.48 ± 0.32	0.34 ± 0.44	3.47 ± 3.08
		TA	0.90 ± 0.03	0.35 ± 0.41	0.15 ± 0.11	9.20 ± 8.03
		PER	0.87 ± 0.06	0.38 ± 0.39	0.19 ± 0.14	8.69 ± 8.70
		EDL	0.79 ± 0.04	0.31 ± 0.35	0.30 ± 0.11	8.96 ± 7.41
		TP	0.85 ± 0.07	0.32 ± 0.41	0.31 ± 0.36	16.07 ± 13.89
	ResNet	GM	0.91 ± 0.04	0.46 ± 0.34	0.13 ± 0.08	3.93 ± 3.10
		GL	0.77 ± 0.13	0.36 ± 0.33	0.66 ± 0.70	4.50 ± 3.99
		SOL	0.84 ± 0.10	0.50 ± 0.30	0.39 ± 0.40	3.43 ± 3.25
		TA	0.88 ± 0.05	0.34 ± 0.41	0.25 ± 0.27	10.68 ± 9.00
		PER	0.84 ± 0.09	0.39 ± 0.35	0.31 ± 0.32	5.12 ± 4.09
		EDL	0.77 ± 0.07	0.28 ± 0.36	0.36 ± 0.16	12.60 ± 10.56
		TP	0.83 ± 0.05	0.33 ± 0.40	0.32 ± 0.30	10.39 ± 8.64
	DenseNet	GM	0.77 ± 0.13	0.31 ± 0.34	0.75 ± 0.66	5.87 ± 4.27
		GL	0.71 ± 0.16	0.27 ± 0.33	0.89 ± 0.58	11.17 ± 11.47
		SOL	0.78 ± 0.12	0.33 ± 0.36	0.70 ± 0.45	8.42 ± 7.78
		TA	0.84 ± 0.07	0.30 ± 0.39	0.24 ± 0.12	19.01 ± 15.56
		PER	0.72 ± 0.14	0.29 ± 0.31	1.11 ± 1.22	7.97 ± 5.50
		EDL	0.68 ± 0.10	0.23 ± 0.32	0.69 ± 0.38	15.24 ± 12.65
		TP	0.80 ± 0.08	0.29 ± 0.38	0.26 ± 0.11	17.75 ± 15.53
Heterogeneous T_het_	U-Net	GM	0.92 ± 0.04	0.83 ± 0.12	0.11 ± 0.08	0.42 ± 0.60
		GL	0.78 ± 0.18	0.73 ± 0.14	0.41 ± 0.45	0.60 ± 0.40
		SOL	0.84 ± 0.13	0.83 ± 0.08	0.40 ± 0.52	0.40 ± 0.35
		TA	0.90 ± 0.04	0.85 ± 0.06	0.15 ± 0.10	0.25 ± 0.17
		PER	0.87 ± 0.08	0.82 ± 0.08	0.21 ± 0.20	0.34 ± 0.30
		EDL	0.77 ± 0.08	0.75 ± 0.10	0.36 ± 0.08	0.45 ± 0.35
		TP	0.86 ± 0.05	0.80 ± 0.06	0.19 ± 0.10	0.31 ± 0.41
	ResNet	GM	0.92 ± 0.04	0.83 ± 0.10	0.16 ± 0.17	0.49 ± 0.44
		GL	0.83 ± 0.10	0.73 ± 0.12	0.32 ± 0.28	0.66 ± 0.44
		SOL	0.85 ± 0.10	0.83 ± 0.07	0.33 ± 0.39	0.36 ± 0.25
		TA	0.90 ± 0.03	0.84 ± 0.05	0.16 ± 0.13	0.26 ± 0.15
		PER	0.86 ± 0.08	0.80 ± 0.09	0.30 ± 0.33	0.41 ± 0.35
		EDL	0.79 ± 0.06	0.73 ± 0.10	0.38 ± 0.24	0.52 ± 0.38
		TP	0.85 ± 0.03	0.79 ± 0.08	0.18 ± 0.06	0.34 ± 0.25
	DenseNet	GM	0.91 ± 0.03	0.83 ± 0.10	0.16 ± 0.14	0.44 ± 0.44
		GL	0.87 ± 0.06	0.78 ± 0.11	0.22 ± 0.15	0.59 ± 0.55
		SOL	0.86 ± 0.08	0.85 ± 0.07	0.35 ± 0.42	0.36 ± 0.32
		TA	0.89 ± 0.05	0.85 ± 0.07	0.22 ± 0.30	0.30 ± 0.25
		PER	0.87 ± 0.07	0.82 ± 0.07	0.26 ± 0.32	0.33 ± 0.25
		EDL	0.78 ± 0.06	0.75 ± 0.10	0.40 ± 0.27	0.55 ± 0.53
		TP	0.86 ± 0.03	0.81 ± 0.84	0.17 ± 0.04	0.31 ± 0.18

EDL = extensor digitorum longus; GL = gastrocnemius lateralis; GM = gastrocnemius medialis; PER = peroneal group; SOL = soleus; TA = tibialis anterior; TP = tibialis posterior.
